# Element Profiling and Probabilistic Health Risk Assessment of Cornelian Cherry Tarhana: The Role of Fermentation and Alternative Flours

**DOI:** 10.1007/s12011-026-05084-8

**Published:** 2026-04-09

**Authors:** Erkan Yalçın, Betül Cındık, Akif Arı, Pelin Ertürk-Arı, Seda Karasu-Yalcin, Eftade O. Gaga

**Affiliations:** 1 Faculty of Engineering, Department of Food Engineering, Bolu Abant Izzet Baysal University, Bolu, Türkiye; 2 Faculty of Engineering, Department of Environmental Engineering, Bolu Abant Izzet Baysal University, Bolu, Türkiye; 3 Faculty of Engineering, Department of Environmental Engineering, Eskişehir Technical University, Eskişehir, Türkiye

**Keywords:** Cornelian Cherry Tarhana, Fermentation, Elements, Clear Flour, Buckwheat Flour, Hull-less Barley Flour

## Abstract

Cornelian cherry tarhana (CCT) is a traditional wheat flour-based powder product used for preparing ready-to-drink soup refreshment in Türkiye. On the other hand, the composition of this food material remains largely uncharacterized regarding its multi-elemental profile and safety. This study provides the first comprehensive elemental characterization of CCT using ICP-MS/MS, uniquely integrating fermentation-driven mineral dynamics with utilization of alternative flours and probabilistic health risk assessment. Our results reveal that fermentation acts as a functional modulator of elemental composition; specifically, clear flour (CF)-based fermented CCT exhibited superior concentrations of Mg and K, which are vital for bone health and cardiovascular regulation, respectively. Conversely, a systematic 12–28% decline in Fe levels post-fermentation was observed likely attributed to adsorption of microorganisms to minerals and the formation of insoluble mineral-phytate complexes during the acidification process. Furthermore, a probabilistic health risk assessment modelled on a once-a-week consumption scenario confirmed that CCT poses no significant carcinogenic or non-carcinogenic risks, with Hazard Index (HI) values remaining well below 1. By establishing this analytical framework, the present research offers a new benchmark for assessing the functional quality and safety of traditional fermented products, providing essential data for both nutritional formulation and regulatory standards.

**Supplementary Information:**

The online version contains supplementary material available at 10.1007/s12011-026-05084-8.

## Introduction

Cornelian cherry tarhana (CCT) is a refined wheat flour-based powder product used for preparing ready-to-drink soup refreshment in Türkiye, mostly produced in province of Bolu and West Black Sea region and has geographical mark. It is also called “sour tarhana” or “kiren tarhana” [1]. Tarhana with cornelian cherry puree is different from the traditional fermented tarhana with its process without fermentation [2]. It is produced by mixing wheat flour, cornelian cherry puree and salt, followed by kneading and drying in a cool place without sun-light and then final sieving from 1 mm sieve in order to get powder form [2, 3]. Its soup is made by mixing the dry product with water, garlic and oil and then boiling for a few minutes.

The traditional fermented tarhana provides high nutritional quality with its proteins, vitamins, and minerals [4]. In addition, fruits contain important amounts of major and minor elements which are needed in the human diets. Fruits also provide much of the recommended dietary allowance (RDA) value for minerals [5]. Cornelian cherry fruit, which is the main ingredient of the CCT, is known with its high antioxidant activity and bioactive compounds. In addition, it has rich mineral composition, including K, Ca, Mg, Na, Fe, Cu, P and Zn. Utilization of cornelian cherry in tarhana enhances nutritional value in terms of bioactive compounds and minerals.

Minerals are essential nutrients which are needed in relatively small quantities-generally less than 100 mg/day [6]. Low mineral availability in staple foods can lead to malnutrition and to physiological pathology, such as osteoporosis, impairment of child growth, and anemia [7]. However, they can be toxic when they are consumed at high concentrations. Macro-elements such as K, S, Mg, Ca, P, and Cl are the main components of organic matter but also have important metabolic functions. Therefore, macro-elements are the main cellular and structural building materials but also take role in osmotic pressure and acid/base regulation. Micro-elements such as B, Co, Cu, Fe, Li, Mo, Mn, Ni, Rb, and Zn participate in the metabolic functions and are the components of enzymes, hormones, vitamins etc. [8, 9]. Heavy metals (Hg, As, Cr, Cu, Ni, Pb, Zn and Cd) may cause deleterious effects on human health due to the ingestion of food grain grown in contaminated soils. For human body, certain heavy metals are essential for the biological systems as structural and catalytic components of proteins and enzymes like Zn and Cu, and others are contaminants such as Cd, As, Hg, Pb, Cr, Ni and so on. However, excessive retention of either kind of heavy metals in the environment imposes health risk for human [10].

In this study, we hypothesize that the incorporation of alternative flours significantly modulates the mineral composition of CCT, and fermentation acts as a functional tool to improve the mineral profile, thereby enhancing overall safety. Consequently, to address how different flour matrices and the fermentation process modify the concentration and risk profile of essential and toxic elements, we produced CCT formulations using various flour types (wheat, buckwheat, clear flour, and wholegrain hull-less barley) under both fermented and non-fermented conditions. By employing advanced ICP-MS/MS analysis, this research aims to provide the first comprehensive elemental baseline for CCT, filling a critical gap in the literature. Finally, to evaluate the safety and nutritional relevance of these products, a probabilistic health risk assessment was performed, based on a realistic once-a-week consumption scenario. This integrated approach provides essential data for the development of scientifically validated functional foods and offers a robust benchmark for future studies on traditional fermented products.

## Materials and Methods

### Materials

Cornelian cherry fruits were obtained from the local farmers in Bolu, Türkiye. They were mashed for obtaining fruit pulp after washing. Refined wheat flour (WF) was provided from the local flour milling company. Buckwheat (*cv. Güneş*) was obtained from Bahri Dağdaş International Agricultural Research Institute, Konya, Türkiye. It was roller-milled (Roller Mill Quadrumat Junior, Brabender GmbH & Co., Duisburg, Germany) for obtaining fine buckwheat flour (BWF). Durum wheat clear flour (CF) as by-product from semolina production was obtained from a pasta factory in Düzce, Türkiye. Hull-less barley (*cv. Özen*) was provided from the Field Crops Central Research Institute (Ankara, Türkiye). It was stone-milled (A700 Genuine Wood, Good Mills Company, Lienz, Austria) in order to produce wholegrain hull-less barley flour (WHBF). Commercial cornelian cherry tarhana (CCCT) and commercial traditional tarhana (CTT) produced with fermentation by mixing wheat flour, some vegetables and yoghurt were purchased from local producers of Bolu, Türkiye.

## Methods

### Cornelian Cherry Tarhana (CCT) Production

The CCT powders were produced using wheat flour (WF), buckwheat flour (BWF), clear flour (CF) and wholegrain hull-less barley flour (WHBF) with or without fermentation process according to Karademir & Yalçın [2] and Bellici et al. [11]. The compositions of different CCT dough were presented in Table 1. Cornelian cherry pulp (P) was mixed (Kitchen Aid, USA) with required amount of flour until obtaining suitable dough consistency, followed by salt addition. The dough mixture was kneaded. Then, it was divided into small pieces, dried in a place without sunlight for 24 h and sieved from 2 mm sieve. It was finally dried without sunlight at room temperature for six days and then sieved from 1 mm sieve. The fermented CCT were produced by applying dough fermentation in a fermentation incubator (Binder, Tuttlingen, Germany) at 25 °C and 60% relative humidity for 72 h. The other production steps were the same as the production of non-fermented samples. In the production of CCT with buckwheat flour (BWF), gluten contamination was prevented by working in separate place. All of the CCT powders (Fig. S1) were produced in duplicate. CCT powders were stored in plastic bags at 4 °C until analysis.

**Table 1:** CCT formulations prepared according to dough consistencies for both Non-fermented (N) and Fermented (F) products

Ingredients(%)	Cornelian Cherry Tarhanas (N-/F-)			
	WF	BWF	CF	WHBF
P	52.7	55.3	58.0	59.4
Salt	5.3	5.5	5.8	5.9
WF	42.0	-	-	-
BWF	-	39.2	-	-
CF	-	-	36.2	-
WHBF	-	-	-	34.7

*P* Cornelian cherry pulp, *WF* Wheat flour, *BWF* Buckwheat flour, *CF* Clear flour,*WHBF* Wholegrain hull-less barley flour

### Determination of Element Contents Using ICP-MS/MS Technique

#### Determination of Element Compositions

Inductively coupled plasma tandem mass spectrometry (ICP-MS/MS: 8800 Triple Quadrupole MS, Agilent Technologies Inc., Santa Clara, CA, USA) was applied for the detection of elemental composition in acid-digested samples according to a previous procedure given by Arı et al. [12]. Detailed analytical validation parameters including instrument linearity, method detection limits (MDL), certified reference material (CRM) recovery rates, and intra-day/inter-day precision metrics are fully documented and verified in the aforementioned study [12]. Further details for the analytical methodology are given in Supplementary Information for the assessed elements in this study. All concentrations were calculated on dry weight basis.

#### Digestion of the Samples

Cornelian cherry tarhana (CCT) samples were first digested in a microwave aided acid digestion unit (Speedwave XPERT, Berghof Products + Instruments GmbH, Eningen, Germany) equipped with a rotor for 10 samples and PTFE digestion vessels as described in the method of Arı et al. [12]. For each type of tarhana powder, around 2 g sample was digested in a mixture of 6.5 mL of deionized water (18.2 MΩ), 2 mL of 35% H_2_O_2_ (Suprapur, Merck KGaA, Darmstadt, Germany) and 1.5 mL of 65% HNO_3_ (Suprapur, Merck KGaA, Darmstadt, Germany). This sample mass was chosen to ensure the representativeness of the complex food matrix while remaining within the operational limits of the microwave digestion system to guarantee complete mineralization and high analytical precision. Double distilled and purified HNO_3_ was used to avoid any elemental interference during the analysis, using an infrared acid purification system (Berghof Distillacid, Berghof Products + Instruments GmbH, Eningen, Germany). The temperature of microwave digester was adjusted to 200 °C within 5 min at 1200 W power and used at this temperature for 15 min. After the isothermal phase, digestion vessels were cooled down to room temperature, and the samples were taken into acid-cleaned volumetric perfluoroalkoxy (PFA) flasks. The samples were diluted to 25 mL with deionized water before analysis.

#### Dietary Risk Assessment

The United States Environmental Protection Agency (USEPA) risk assessment guidelines were used in determination of carcinogenic and non-carcinogenic health risk assessments associated with heavy metal ingestion through the consumption of CCT samples [13]. Using the average daily dose (*ADD*) amounts together with pertinent chronic oral reference doses (*RfD*) and cancer slope factors (*SF*) documented by the authorities, additional computations were performed for the various carcinogenic and non-carcinogenic risks. The *ADDs* of heavy metals via consumption of CCT samples were calculated using the following Eq. 1 [14]:

1 $$ADD_i=\:\frac{Ci\times\:IR\times\:\varphi\:i\times\:EF\times\:ED}{BW\times\:AT}$$

Where *ADD*_*i*_ is the average daily intake of metal *i* (mg/kg-day), *C*_*i*_ is the concentration of the *i*^*th*^ metal in tarhana sample (mg/kg), *IR* is the ingestion rate of tarhana sample (0.1 kg/day), φ_i_ is the bioaccessibility rate of metal *i* (%), *EF* is the frequency of exposure (52 days/year, representing a once-a-week consumption), *ED* is the exposure duration (70 years for a lifetime), *BW* is the body weight (66.4 ± 8.8 kg), and *AT* is the averaging time (70 × 350 days/year for carcinogens and *AT* = *ED* × *EF* for non-carcinogens) [15]. The bioaccessibility rates for Fe, Mn, Zn, Cr, As, Cd, and Pb were 65%, 79%, 65%, 55%, 75%, 75%, and 33%, respectively [16].

Non-carcinogenic and carcinogenic health risks aroused via ingestion of food containing heavy metals were assessed through hazard quotients (*HQs*) and incremental lifetime cancer risk (*ILCR*). The 7 heavy metals (As, Pb, Cd, Cr, Fe, Mn, Zn) had non-carcinogenic health risks, while As, Cd, and Cr had carcinogenic risks. Correspondingly, *HQ*s were estimated dividing the ADD amounts by the *RfDs* of individual heavy metals indicated in Eq. (2), and the carcinogenic *SFs* were multiplied by *ADDs* for the estimation of *ILCRs* using Eq. (3):

2 $$HQ_i=\:\frac{ADDi}{RfDi}$$

3 $$ILCR_{i}=ADD_{i\times}SF_{i}$$

The reference toxicological values (*RfDs* and *SFs*) were carefully gathered from Exposure Factors Handbook of USEPA [17]. The oral *RfD* values for As, Cd, Cr, Fe, Mn, Pb, and Zn were set as 3 × 10^− 4^, 1 × 10^− 3^, 9 × 10^− 4^, 0.7, 0.14, 3.7 × 10^− 3^, and 0.3 (mg/kg-day), respectively. Cancer slope factors for As, Cd, and Cr were 1.5, 0.6, and 0.50 (kg-day/mg), respectively. In contrast to other toxic and carcinogenic elements, the *RfD* and *SF* values for Cr were primarily defined for its hexavalent form, Cr(VI). Since no isotopic speciation was performed during the elemental analysis in this study. A worst-case scenario was adopted by assuming the Cr(VI) concentration to be one-seventh (1/7) of the measured total Cr concentration. Consequently, health risk characterizations were conducted based on these hypothetical Cr(VI) levels [18]. The estimated *HQ* values above 1.0 indicate that there is a potential non-carcinogenic risk to the human body from heavy metal intake via consumption of tarhana sample, and values below 1.0 indicates no harm. An *ILCR* higher than 10^− 4^ indicates that there is a significant risk of cancer due to heavy metal exposure through tarhana consumption. The *ILCR* values between 10^− 4^ and 10^− 6^ were acceptable and indicate no significant risk for cancer [14, 19]. The exposure scenario was established based on a “once-a-week” consumption assumption. This frequency is derived from national dietary intake patterns, which categorize traditional tarhana as a regular, yet not necessarily daily, soup component in the Turkish diet. Given that consumers alternate between various traditional soups (such as lentil, yogurt, or vegetable-based soups) throughout the week, a weekly consumption rate provides a balanced and realistic estimation of chronic exposure, preventing both overestimation and underestimation of the associated health risks.

### Statistics

All analysis was done at least quadruplicate and more. The mean values and standard deviations were recorded. One-way analysis of variance (ANOVA) was used for data analysis in SPSS (Version 20.0 for Windows) statistical programme. For determining the differences among means at 95% confidence level (*p* < 0.05), the Duncan’s multiple range tests were used. Due to the several parameters randomly affecting the exposure analyses, the uniformity of the results should be controlled by statistical techniques in risk assessment studies [20]. Crystal Ball software (Oracle Inc., USA, version 11.1.2.4) was used to perform Monte Carlo random sampling method simulations, each with 1000 iterations (at 95% confidence interval) for health risk assessment calculations to evaluate the uncertainty and the sensitivity of the input parameters.

## Results and Discussion

Macro- and micro (trace)-element contents (mg/g, µg/g, respectively) were detected for the used flours and CCT products. The results obtained for the flours from different raw materials were shown in Table 2. The highest amounts of Mg (0.85 mg/g), K (3.18 mg/g), S (1.52 mg/g) and Ca (0.14 mg/g) were detected in clear flour (*p* < 0.05). Mg (0.16 mg/g), K (0.80 mg/g) and Ca (0.06 mg/g) concentrations were at lowest levels in wheat flour. The highest amount of Na (0.35 mg/g) was obtained in wholegrain hull-less barley flour (*p* < 0.05). Buckwheat flour was found to contain 1.03 mg/g K and 0.80 mg/g S while Ca and Na were at very low amounts. Micro (trace)-elements of Li (0.29 µg/g), B (1.44 µg/g), Al (23.09 µg/g) and Sm (5.45 µg/g) were at highest levels in buckwheat flour (*p* < 0.05). Clear flour was rich in Mn (23.23 µg/g) and Rb (2.23 µg/g, *p* < 0.05). Wholegrain hull-less barley flour was prominent with its Sr (2.41 µg/g) and Mo (0.54 µg/g) contents, while wheat flour contained the highest amount of In (32.36 µg/g, *p* < 0.05).

**Table 2:** Macro- and micro (trace)-element contents of raw materials used in the production of CCT powders

Macro-Elements (mg/g)^*^	WF	WHBF	CF	BWF
Na	0.30 ± 0.012^c^	0.35 ± 0.014^a^	0.33 ± 0.009^b^	0.27 ± 0.013^d^
Mg	0.16 ± 0.002^d^	0.69 ± 0.012^b^	0.85 ± 0.012^a^	0.45 ± 0.017^c^
K	0.80 ± 0.004^d^	2.62 ± 0.098^b^	3.18 ± 0.057^a^	1.03 ± 0.060^c^
S	1.15 ± 0.059^c^	1.35 ± 0.018^b^	1.52 ± 0.023^a^	0.80 ± 0.008^d^
Ca	0.06 ± 0.002^c^	0.11 ± 0.005^b^	0.14 ± 0.003^a^	0.11 ± 0.002^b^

Micro (Trace)-Elements(µg/g)**	WF	WHBF	CF	BWF
Li	0.02±0,01^c^	0.16±0.01^b^	0.02±0.01^c^	0.29±0.02^a^
B	0.28±0,04^d^	0.81±0.03^c^	0.92±0.05^b^	1.44±0.13^a^
Al	1.96±0,43^d^	12.18±0.72^c^	16.27±0.78^b^	23.09±1.52^a^
Mn	3.13±0,09^c^	10.35±0.40^b^	23.23±1.11^a^	2.19±0.02^d^
Rb	0.44±0,01^d^	0.85±0.01^c^	2.23±0.10^a^	1.09±0.03^b^
Sr	0.86±0,02^d^	2.41±0.03^a^	1.73±0.02^b^	1.06±0.04^c^
Mo	0.20±0,01^d^	0.54±0.02^a^	0.39±0.01^b^	0.24±0.02^c^
In	32.36±0,88^a^	30.47±1.45^b^	13.31±0.77^d^	28.28±1.08^c^
Sm	0.00	3.56±0.11^b^	0.00	5.45±0.19^a^

Mean±standard deviation (*n=4; **n=5). All results were on dry weight basis. Means with the different letters within each line are significantly different (*p*<0.05). *WF* Wheat flour, *WHBF* Wholegrain hull-less barley flour, *CF* Clear flour, *BWF* Buckwheat flour

The essential and toxic heavy-metal contents of different flours used in the production of CTT powders (µg/g of sample) were shown in Table 3. Clear flour was rich in Cr (0.38 µg/g), Fe (36.0 µg/g), Ni (0.88 µg/g), Cu (2.0 µg/g), Zn (32.0 µg/g) and Ti (1.56 µg/g, *p* < 0.05). The highest amounts of Pb (0.094 µg/g) and Sn (34.4 µg/g) were obtained in wheat flour which contained Cr, Fe, Cu, Zn and Ti at lowest amounts. Wholegrain hull-less barley flour and buckwheat flour were also rich in Fe and Sn.

**Table 3:** Essential and toxic heavy metal contents of raw materials used in the production of CCT powders (µg/g of sample)

		WF	WHBF	CF	BWF
Essential Elements (µg/g)	Cu	0.5 ± 0.02^d^	1.4 ± 0.15^b^	2.0 ± 0.07^a^	0.9 ± 0.03^c^
	Fe	7.5 ± 0.58^c^	26.6 ± 0.75^b^	36.0 ± 1.74^a^	26.7 ± 0.84^b^
	Zn	3.0 ± 0.08^d^	10.6 ± 0.80^b^	32.0 ± 1.27^a^	4.6 ± 0.15^c^
Toxic Elements (µg/g)	Ni	0.81 ± 0.019^c^	0.53 ± 0.027^d^	0.88 ± 0.017^a^	0.84 ± 0.012^b^
	Cr	0.33 ± 0.006^d^	0.34 ± 0.004^c^	0.38 ± 0.009^a^	0.36 ± 0.006^b^
	Sn	34.4 ± 0.80^a^	34.1 ± 0.68^a^	15.8 ± 1.10^c^	32.6 ± 1.10^b^
	Pb	0.094 ± 0.002^a^	0.077 ± 0.003^c^	0.085 ± 0.001^b^	0.083 ± 0.002^b^
	Ti	0.11 ± 0.010^d^	1.23 ± 0.065^b^	1.56 ± 0.046^a^	0.39 ± 0.013^c^

Mean±standard deviation (*n* = 5). All results were on dry weight basis. Means with the different letters within each *line* are significantly different (*p* < 0.05). *WF* Wheat flour, *WHBF* Wholegrain hull-less barley flour, *CF* Clear flour, *BWF* Buckwheat flour

Macro-element contents (mg/g) of CCT powders (fermented and non-fermented) in comparison with commercial tarhana samples were shown in Table 4. Calcium concentration (0.32 mg/g) of commercial traditional tarhana (CTT) was high with a notable difference which was probably due to utilization of yoghurt in its production. High Ca concentration of traditional tarhana was also demonstrated in some other studies. Ertas [21] reported that Ca contents of traditional tarhana produced from different flours changed between 58.98 and 214.09 mg/100 g. Amount of this element was also reported as 119.2 mg/100g [22] and 1411.9 ppm [4] in traditional tarhana including yoghurt in its formulation. Sodium and K contents of produced CCT were higher than the commercial cornelian cherry tarhana (CCCT) and CTT (*p* < 0.05). Potassium and Na have critical roles in providing fluid balance in the body and regulating blood pressure. In addition, K is also known to contribute to the utilization of Fe, regulation of acid-base balance and enhance muscle functions and nerve actions [23]. The CCT produced from different raw materials could be essential in preventing hypertension and muscle paralysis. Especially potassium contents of CCT produced from clear flour and wholegrain hull-less barley flour were higher than the reported values of 3.99 mg/100g [21] and 3393.5 ppm [4] for traditional tarhana, probably due to their raw materials rich in these elements. Amount of Mg in CCT powders was also higher than the control tarhanas, except for the CCT produced with wheat flour and without fermentation. The highest amount of Mg (0.59 mg/g) was obtained in fermented CCT with clear flour. This result was similar to the Mg concentration (546.8 ppm) of traditional tarhana reported by Isik and Yapar [4] which was increased by addition of tomato seed. Magnesium is reported as a critical mineral for bone formation, hormonal activation and secretion and energy production. Magnesium takes role as a cofactor for many enzymes besides its critical roles in maintaining body osmotic balance and glucose homeostasis [23]. Fermented CCT produced with clear flour was prominent with its high Mg, K and S contents, which can be expected to transfer from clear flour as a source of macro-elements. Fermentation caused a significant increase (*p* < 0.05) in Na contents of CCT produced by using clear flour and buckwheat flour. Magnesium concentration significantly (*p* < 0.05) decreased after fermentation in CCT produced with hull-less barley flour, but increased (*p* < 0.05) in CCT with buckwheat flour. Calcium, K and S contents also significantly increased (*p* < 0.05) in CCT powders produced with buckwheat flour and clear flour after fermentation. Sukhikh et al. [24] reported that Na, K, Ca and Mg contents of soybean meal increased by fermentation, similar to our results. It is thought that enhancement of macro-element composition of CCT by fermentation is promising for nutrition quality and gaining a new perspective for fermented foods.

**Table 4:** Macro-element contents of Fermented (F-) and Non-fermented (N-) CCT powders in comparison with commercial tarhana samples

CCT Samples	Na (mg/g)	Mg (mg/g)	K (mg/g)	S (mg/g)	Ca (mg/g)
N-WF	18.5 ± 0.00^d^	0.19 ± 0.005^g^	2.55 ± 0.008^f^	1.32 ± 0.019^e^	0.22 ± 0.004^f^
N-WHBF	21.9 ± 0.31^a^	0.57 ± 0.015^b^	4.42 ± 0.004^b^	1.36 ± 0.025^d^	0.27 ± 0.004^d^
N-CF	19.9 ± 0.35^c^	0.57 ± 0.016^ab^	4.17 ± 0.046^c^	1.29 ± 0.020^e^	0.27 ± 0.005^d^
N-BWF	15.3 ± 0.13^e^	0.37 ± 0.006^e^	2.55 ± 0.075^f^	0.90 ± 0.009^h^	0.23 ± 0.001^e^
F-WF	18.3 ± 0.34^d^	0.18 ± 0.008^g^	2.90 ± 0.052^e^	1.24 ± 0.022^f^	0.22 ± 0.004^f^
F-WHBF	19.6 ± 0.41^c^	0.49 ± 0.004^c^	4.21 ± 0.036^c^	1.29 ± 0.012^e^	0.28 ± 0.006^c^
F-CF	20.8 ± 0.65^b^	0.59 ± 0.013^a^	4.88 ± 0.024^a^	1.47 ± 0.019^b^	0.31 ± 0.003^b^
F-BWF	18.8 ± 0.51^d^	0.45 ± 0.018^d^	3.21 ± 0.043^d^	0.96 ± 0.009^g^	0.27 ± 0.003^d^
CCCT^a^	8.3 ± 0.59^g^	0.25 ± 0.016^f^	2.07 ± 0.074^g^	1.43 ± 0.047^c^	0.18 ± 0.005^h^
CTT^b^	11.4 ± 0.66^f^	0.26 ± 0.11^f^	1.68 ± 0.096^h^	1.79 ± 0.032^a^	0.32 ± 0.005^a^

Mean±standard deviation (*n* = 5). All results were on dry weight basis. Means with the different letters within each *column* are significantly different (*p* < 0.05). ^a^Commercial Cornellian Cherry Tarhana, ^b^Commercial Traditional Tarhana. *WF* Wheat flour, *WHBF* Wholegrain hull-less barley flour, *CF* Clear flour, *BWF* Buckwheat flour

Micro (trace)-element contents (µg/g) of CCT powders (fermented and non-fermented) in comparison with commercial tarhana samples were shown in Table 5. Lithium and B contents of the produced CCT powders were lower than the commercial CCT. Aluminium concentrations in all CCT products were higher than the commercial CCT, except the product with wheat flour. Aluminium concentrations were higher especially in clear flour and buckwheat flour-based CCT, related to their raw materials rich in Al. Tekgül et al. [22] reported that traditional tarhana contained 17.3 ppm Al which was increased by supplementation of wheat germ. This value is higher than Al content of most of the produced CCT in this study. Aluminium is found in the aluminium silicate minerals in soil, and the soluble forms of Al can influence biological systems. The mononuclear Al^3+^ species and Al_13_ are considered as the most toxic forms [25]. Indium contents of the produced CCT powders were also higher than the commercial tarhanas, except the non-fermented and fermented CCT with WHBF. Manganese concentrations of CCT with clear flour were higher than the commercial tarhanas and the other CCT samples (*p* < 0.05). It was attributed to considerably high amount of Mn in clear flour when compared to other flours (Table 2). However, Mn concentrations of CCT with wheat flour and buckwheat flour in both productions were lower than the commercial tarhanas (*p* < 0.05). In carbohydrate, protein and fat metabolism, manganese takes important roles as a cofactor of many metabolic enzymes [23].

**Table 5:** Micro (trace)-element contents of Fermented (F-) and Non-fermented (N-) CCT powders in comparison with commercial tarhana samples

CCT Samples	Li (µg/g)	B (µg/g)^c^	Al (µg/g)	Mn (µg/g)	Rb (µg/g)	Sr (µg/g)	Mo (µg/g)	In (µg/g)^a^	Sm (µg/g)
N-WF	0.00	0.69 ± 0.013^e^	4.1 ± 0.33^g^	2.6 ± 0.02^f^	0.7 ± 0.01^f^	3.3 ± 0.11^ef^	0.18 ± 0.007^g^	52.2 ± 0.96^b^	1.5 ± 0.13^d^
N-WHBF	0.24 ± 0.027^b^	0.88 ± 0.019^b^	11.5 ± 0.62^c^	7.5 ± 0.07^c^	1.1 ± 0.04^d^	4.5 ± 0.23^a^	0.37 ± 0.018^a^	40.7 ± 0.69^d^	0.7 ± 0.09^e^
N-CF	0.15 ± 0.025^d^	0.59 ± 0.012^f^	12.0 ± 1.07^c^	14.2 ± 0.38^b^	1.6 ± 0.14^b^	3.9 ± 0.21^d^	0.23 ± 0.007^c^	51.7 ± 1.28^b^	0.7 ± 0.08^e^
N-BWF	0.21 ± 0.026^c^	0.71 ± 0.006^e^	21.0 ± 1.21^a^	1.9 ± 0.09^g^	1.2 ± 0.08^c^	3.1 ± 0.08^g^	0.18 ± 0.012^g^	52.4 ± 0.84^b^	4.1 ± 0.26^a^
F-WF	0.00	0.78 ± 0.015^d^	4.1 ± 0.10^g^	2.5 ± 0.01^f^	0.8 ± 0.02^e^	3.2 ± 0.06f^g^	0.17 ± 0.010^g^	54.7 ± 0.54^a^	0.7 ± 0.08^e^
F-WHBF	0.22 ± 0.028^bc^	0.71 ± 0.020^e^	8.5 ± 0.58^d^	6.6 ± 0.06^d^	1.1 ± 0.04^d^	4.4 ± 0.10^b^	0.34 ± 0.013^b^	36.9 ± 0.48^e^	2.1 ± 0.07^c^
F-CF	0.11 ± 0.015^e^	0.51 ± 0.008^g^	12.8 ± 0.81^b^	14.8 ± 0.52^a^	2.2 ± 0.15^a^	4.0 ± 0.11^c^	0.22 ± 0.006^cd^	52.3 ± 2.02^b^	2.1 ± 0.12^c^
F-BWF	0.23 ± 0.018^bc^	0.79 ± 0.013^d^	20.5 ± 0.88^a^	2.0 ± 0.10^g^	1.3 ± 0.13^c^	3.3 ± 0.14^ef^	0.21 ± 0.009^de^	51.9 ± 0.49^b^	0.00
CCCT^b^	0.58 ± 0.039^a^	1.14 ± 0.006^a^	5.1 ± 0.40^f^	4.3 ± 0.08^e^	1.1 ± 0.04^d^	3.4 ± 0.06^e^	0.20 ± 0.009^e^	44.6 ± 0.52^c^	2.6 ± 0.18^b^
CTT^c^	0.14 ± 0.013^d^	0.86 ± 0.045^c^	6.2 ± 0.23^e^	4.4 ± 0.04^e^	1.0 ± 0.04^d^	3.1 ± 0.03^g^	0.19 ± 0.007^f^	45.4 ± 1.05^c^	0.6 ± 0.01^e^

Mean±standard deviation (*n* = 10; ^a^*n*=5). All results were on dry weight basis. Means with the different letters within each *column* are significantly different (*p* < 0.05). ^b^Commercial Cornellian Cherry Tarhana (*n* = 5), ^c^Commercial Traditional Tarhana (*n* = 5). *WF* Wheat flour, *WHBF* Wholegrain hull-less barley flour, *CF* Clear flour, *BWF* Buckwheat flour

Boron, Rb and In contents of CCT with wheat flour increased after fermentation. In CCT with hull-less barley flour, Li, B, Al, Mn, Sr, Mo and In concentrations decreased, but Sm increased by fermentation process. In CCT with clear flour, fermentation caused an increase in all micro (trace)-elements except for Li, B and Mo. Lithium, B, Sr and Mo contents of CCT with buckwheat flour significantly increased (*p* < 0.05) and the concentrations of Al, Mn, Ru and In did not change significantly (*p* > 0.05) after fermentation. However, a drastic decrease was detected in Sm content (Table 5).

The essential and toxic heavy-metal contents of CCT products (fermented and non-fermented) in comparison with commercial tarhana samples were shown in Table 6. The Fe content of non-fermented clear flour-based CCT was considerably high, which was thought to be a result of clear flour composition (Table 2). Iron contents of all produced CCT were higher than that of commercial CCT and traditional tarhanas. Produced CCT in this study could be evaluated as a rich source of Fe when compared to reported results about traditional tarhana. Ertaş [21] reported the Fe content of traditional tarhana produced with wheat flour as 2.84 mg/100 g. In another study, amount of Fe in traditional tarhana was found as 23.3 ppm and it was increased to 48.9 ppm after tomato seed supplementation [4]. Iron is an essential element important in the formation of blood and transport of oxygen throughout the body as a component of haemoglobin. It has also essential roles as a component of protein and some drug metabolizing enzymes requiring cytochrome P450 [23]. The high Fe content of CCT samples can be evaluated as promising for possible health benefits against anaemia, dysfunction immune system and other diseases associated with iron deficiency. Iron concentrations decreased by fermentation process for all products (*p* < 0.05). The decrease of Fe in CCT with WHBF and CF was very distinct. In CCT with wheat flour, concentrations of Ni and Ti increased, and Zn decreased after fermentation (*p* < 0.05). Fermentation caused a decrease in all of the essential and toxic heavy metal concentrations in CCT with WHBF except for Ti. Chromium, Ni, Cu, Sn, Pb and Ti concentrations increased after fermentation in clear flour-based CCT. In buckwheat flour-based CCT, increase in Cr and Ti concentrations was observed, while the amounts of Fe, Ni and Sn decreased (*p* < 0.05). Although being a toxic element, chromium (Cr) takes role in the metabolism of proteins, carbohydrates and lipids, while the most important function is its action on glucose uptake by cells. This element has also been related to reductions in serum total cholesterol, LDL-cholesterol, and triglyceride levels [26]. However, for some heavy metals including Cr, Ni, Sn, Pb and Ti, decreasing by fermentation should be evaluated as essential according to their health hazards when exist in high concentrations. Toxic elements like Ni can cause undesirable effects in humans according to amount of intake. Plants and crops cultivated in contaminated soil can be the source of toxic elements. Toxic metals may accumulate in vital organs (such as liver, brain, kidney, and heart) and damage their normal functioning [27]. The heavy metal Pb is ubiquitous in the environment and is present at low concentration in most of foods. It is a physiological and neurological toxin, and has carcinogenic effect in humans [28]. Plants may be contaminated by Pb as a result of industrial gas effluent and vehicle traffic, as well as sewage sludges and other wastes in agricultural lands in certain areas. Processing and storage are also critical steps for lead contamination to food. These two conditions are the main reasons for enhanced Pb intake via foodstuffs [28]. Nickel plays an essential role in the growth of all living organisms. However, it is also potential food safety hazard causing a risk for human health, like Ni allergy in sensitive humans by prolonged dermal contact. The production, processing and packaging stages of foods are also the subject of Ni contamination [29]. Amount of Sn was high in non-fermented wheat flour-based CCT, probably due to the raw material rich in Sn. Tin has potential toxicity in food which depends on the ingested amount and also on pH, valence, extent of adsorption and solubility [30]. The Sn exists in two forms, like inorganic Sn (essentially non-toxic) and organic Sn (toxic compound) owning at least one Sn–C bond for exhibiting its toxicity. The inorganic Sn in food is originating from the inside of a tinplate can and it is converted into the organic form [30].

**Table 6:** Essential and toxic heavy metal contents of Fermented (F-) and Non-fermented (N-) CCT powders in comparison with commercial tarhana samples

Essential Elements (µg/g)				Toxic Elements (µg/g)				
CCT Samples	Cu (µg/g)	Fe (µg/g)	Zn (µg/g)	Ni (µg/g)	Cr (µg/g)	Sn (µg/g)	Pb (µg/g)	Ti (µg/g)
N-WF	0.44 ± 0.020^f^	45.5 ± 2.28^c^	5.8 ± 0.23^d^	0.41 ± 0.014^f^	0.37 ± 0.008^d^	66.6 ± 3.15^ab^	0.078 ± 0.014^abc^	0.18 ± 0.009^h^
N-WHBF	1.22 ± 0.156^b^	59.5 ± 2.75^b^	8.1 ± 0.20^c^	0.51 ± 0.015^d^	0.42 ± 0.006^a^	49.4 ± 2.22^e^	0.068 ± 0.006^c^	1.09 ± 0.079^b^
N-CF	1.21 ± 0.059^b^	105.0 ± 7.85^a^	16.3 ± 1.29^a^	0.54 ± 0.016^cd^	0.37 ± 0.009^d^	54.9 ± 3.11^d^	0.079 ± 0.008^ab^	0.86 ± 0.031^c^
N-BWF	0.65 ± 0.024^c^	31.0 ± 2.35^e^	3.2 ± 0.15^f^	0.94 ± 0.049^a^	0.39 ± 0.007^c^	60.5 ± 2.35^c^	0.077 ± 0.008^abc^	0.51 ± 0.016^e^
F-WF	0.50 ± 0.019^ef^	35.1 ± 1.90^d^	3.1 ± 0.16^f^	0.62 ± 0.025^b^	0.37 ± 0.008^d^	68.1 ± 2.33^a^	0.078 ± 0.005^abc^	0.46 ± 0.017^f^
F-WHBF	1.21 ± 0.142^b^	25.0 ± 1.39^f^	7.7 ± 0.19^c^	0.37 ± 0.021^g^	0.32 ± 0.006^f^	40.4 ± 1.04^f^	0.085 ± 0.004^a^	1.14 ± 0.064^b^
F-CF	1.32 ± 0.037^a^	43.8 ± 3.30^c^	14.7 ± 0.49^b^	0.62 ± 0.024^b^	0.40 ± 0.009^b^	65.1 ± 3.26^b^	0.082 ± 0.014^a^	1.20 ± 0.078^a^
F-BWF	0.66 ± 0.028^c^	24.8 ± 1.56^f^	3.2 ± 0.16^f^	0.55 ± 0.040^c^	0.41 ± 0.007^a^	54.4 ± 2.44^d^	0.080 ± 0.013^ab^	0.58 ± 0.022^d^
CCCT^a^	0.61 ± 0.019^cd^	10.6 ± 0.64^g^	4.2 ± 0.11^e^	0.43 ± 0.012^e^	0.36 ± 0.008^e^	48.5 ± 1.81^e^	0.070 ± 0.002^bc^	0.29 ± 0.008^g^
CTT^b^	0.57 ± 0.011^de^	11.1 ± 0.30^g^	6.0 ± 0.10^d^	0.45 ± 0.034^e^	0.33 ± 0.005^f^	56.5 ± 1.20^d^	0.077 ± 0.002^abc^	0.87 ± 0.030^c^

Mean±standard deviation, (*n* = 10). All results were on dry weight basis. Means with the different letters within each *column* are significantly different (*p* < 0.05). ^a^Commercial Cornellian Cherry Tarhana (*n* = 5), ^b^Commercial Traditional Tarhana (*n* = 5). *WF* Wheat flour, *WHBF* Wholegrain hull-less barley flour, *CF* Clear flour, *BWF* Buckwheat flour

Utilization of fermentation process resulted in decreasing of some element concentrations in the product while some of them increased. Both effects are associated with metabolic activities of the fermenting microorganisms. Microbiota of fermented CCT was investigated in our previous study [11]. It was reported that microflora of CCT powders was mainly composed of certain yeasts species which were *Hanseniaspora uvarum*,* Saccharomyces cerevisiae*,* Torulaspora delbrueckii*,* Candida krusei*,* Candida lusitaniae*,* Metschnikowia pulcherrima*,* Wickerhamomyces anomalus*,* Candida kefyr*,* Cyberlindnera fabianii* and *Candida parapsilosis*. Besides, *Lactobacillus reuteri* and *Enterococcus* spp were also isolated as lactic acid bacteria. Initial yeast counts of CCT were recorded between 4.46 and 5.62 log (cfu/mL) at the beginning, while they were 6.24–6.71 log (cfu/mL) at the end of the fermentation. Microorganisms taking role in fermentation process can increase mineral solubility, promoting their release from the food matrix. These microorganisms produce organic acids and reduce the pH which causes the breakdown of compounds binding minerals and heavy metals within the food [31]. Increase in element contents of CCT products after fermentation can be attributed to its rich microbiota which could hydrolyse the metal–phytate complexes to release free minerals for use and losses in dry matter [32]. In addition, breakdown of complex chelated compounds in the fermenting medium leading to an improved synthesis of minerals could cause an increase in mineral content. However, the pH drop by microbial activity can also induce an environment where certain minerals become less soluble, impeding their release from the food matrix and resulting in a decrease in their concentration [31]. In addition, some elements can be utilized by fermenting microorganisms for physiological and metabolic activities [32]. Kiczorowski et al. [33] reported that removal of heavy metals was relatively difficult via culinary processing or during preparation of animal feed. Their study demonstrated that Cu, Pb, and Cd levels decreased after fermentation of vegetables. It was explained as a result of metal binding to the cell walls of fermentative bacteria and fungi. The carboxyl, amino, hydroxyl, phosphate, and sulfhydryl groups present in the external structures of microorganisms were reported to involve in heavy metal chelation. It was revealed that heavy metal ions were adsorbed via complexation with negatively charged reaction sites on the cell surface.

In this study, the composition of the CCT powders was examined in terms of their element concentrations. Furthermore, considering that plant-based foods may contain trace amounts of toxic heavy metals due to environmental factors [34, 35], heavy metal analyses were also performed on the samples. To assess the potential health effects of toxic heavy metals, carcinogenic risks were evaluated using the ingestion risk assessment method recommended by the USEPA. This approach aims to determine whether the detected concentrations pose any risk to human health. The findings showed that the essential nutrients contained in CCT powders contributed positively to daily nutrition, while heavy metal levels remained well below acceptable limits. Accordingly, the calculated *HQ* and *ILCR* values confirmed that regular consumption of the product could not pose any health risks and demonstrated that CCT powders were safe for consumption.

**Fig. 1:**
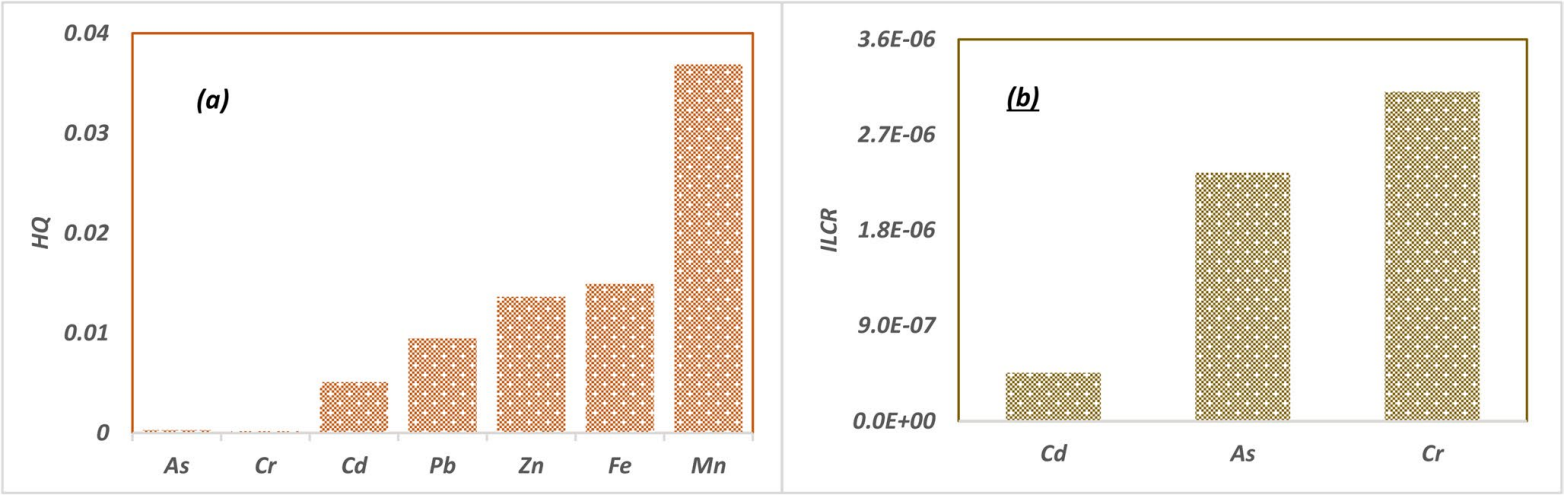
Health risk assessments in means of the individual *HQ*s (**a**) and *ILCR*s (**b**)

**Fig. 2:**
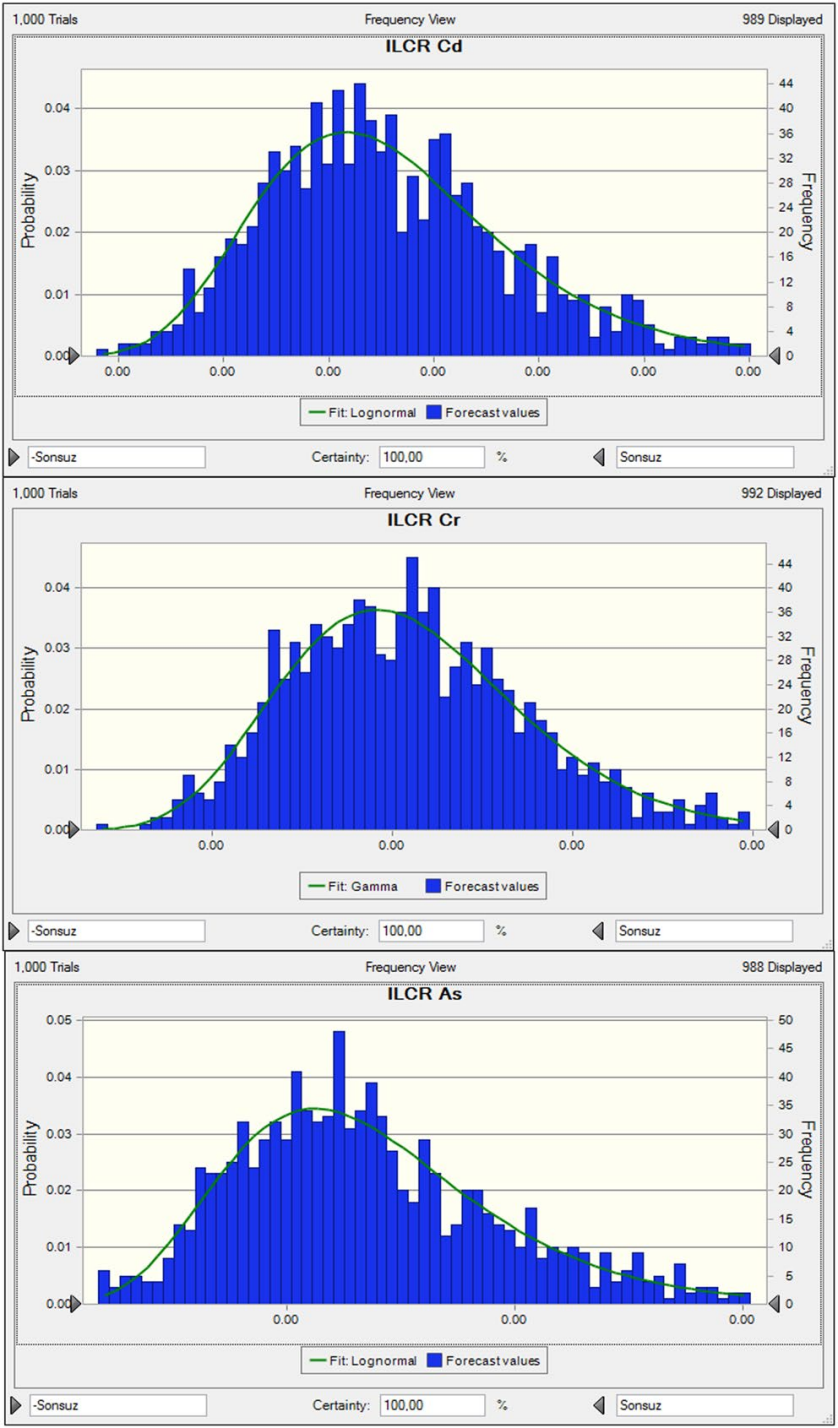
Probability distributions for *ILCR* estimated using random input variables after 1000 iterations

The ADD values ranged from 5.1 × 10^− 6^ (Cd) to 1.04 × 10^− 2^ (Fe) mg/kg-day. Accordingly, the calculated *HQ* values were ranked as follows: Cr < As< Cd < Pb< Zn < Fe< Mn **(**Fig. 1a**)**. On the other hand, neither the individual *HQ*s nor the total *HQ* levels of the seven heavy metals exceeded the threshold of 1.0 established for the occurrence of non-carcinogenic health effects [36], and the total *HQ* was 0.08. Similarly, *ILCR* values were found below the critical value of 10^− 4^, indicating an insignificant potential for additional risk of cancer due to the ingestion of carcinogenic heavy metals via produced CCT powders (Fig. 1b). The highest *ILCR* was calculated for Cr (3.11 × 10^− 6^), and the lowest value was for Cd (4.56 × 10^− 7^). A sensitivity analysis showed that the estimated *ILCR*s were mostly dependent on the concentration of the carcinogenic metals in CCT powders (24.7%, 28.7%, and 31.3% for As, Cd, and Cr, respectively), followed by the body weight (-24.2% to -20.7%), and the ingestion rate (16.9% to 23.4%). Besides, according to the probability distributions of the carcinogenic risks calculated for As, Cd, and Cr (Fig. 2), the critical value of 10^− 4^ was not being exceeded for any exposure to these three elements, and the estimated probability of this value was 0% among 1000 projections, and showed insignificant contribution to overall risk.

## Conclusion

Element composition of CCT samples was determined for the first time. CCT was demonstrated as a good source of elements especially for K, Mg, Fe and Mn. Utilization of different flours changed the element contents in the products. Although fermentation process is not applied in traditional CCT production, fermentation affected most of the element concentrations in the produced CCT products in this research. It was demonstrated that increase or decrease was observed in certain element amounts according to metabolic activities of the microorganisms during fermentation. Essential results were obtained by fermentation process like increasing some macro-elements or decreasing certain toxic heavy metals. In addition, the amount of toxic and/or carcinogenic heavy metals in produced CCT powders presented no significant health risks in terms of carcinogenic and non-carcinogenic risk assessments, indicating that CCT powders were totally safe and ready-to-drink soup nourishments. The bioavailability of macro- and micro (trace)- elements and essential heavy metals after in vitro digestion of fermented CCT powders in cooked forms, and effect of storage after fermentation on element composition can be further investigated.

## Supplementary Information

Below is the link to the electronic supplementary material.

Supplementary Material 1.

## Data Availability

The data sets are available from the corresponding author on reasonable request.
