# Supplementary material for: Element Profiling and Probabilistic Health Risk Assessment of Cornelian Cherry Tarhana: The Role of Fermentation and Alternative Flours
Source: Biol Trace Elem Res. 2026 Apr 9;204(8):5955–65. doi: 10.1007/s12011-026-05084-8 (PMC13369210; doi:10.1007/s12011-026-05084-8)
Supplement: Supplementary file 1 — Supplementary Material 1. [file 12011_2026_5084_MOESM1_ESM.docx]

***Supplementary Information***

**Element Profiling and Probabilistic Health Risk Assessment of Cornelian Cherry Tarhana: The Role of Fermentation and Alternative Flours**

**Erkan Yalçın^1^** ⸱ **Betül Cındık^1^** **⸱ Akif Arı^2^ ⸱ Pelin Ertürk-Arı^2^ ⸱ Seda Karasu-Yalcin^1^ ⸱ Eftade O. Gaga^3^**

^1^Bolu Abant Izzet Baysal University, Faculty of Engineering, Department of Food Engineering, Bolu, Türkiye

^2^Bolu Abant Izzet Baysal University, Faculty of Engineering, Department of Environmental Engineering, Bolu, Türkiye

^3^Eskişehir Technical University, Faculty of Engineering, Department of Environmental Engineering, Eskişehir, Türkiye

**Corresponding author:** Erkan Yalçın

**E-mail:** yalcin_e@ibu.edu.tr

Erkan Yalçın ORCID: 0000-0002-7417-9088

Betül Cındık ORCID: 0000-0001-9765-9861

Akif Arı ORCID: 0000-0003-3870-8779

Pelin Ertürk Arı ORCID: 0000-0001-6508-6042

Seda Karasu-Yalcin ORCID: 0000-0003-0438-1130

Eftade O. Gaga ORCID: 0000-0003-3182-9340

**
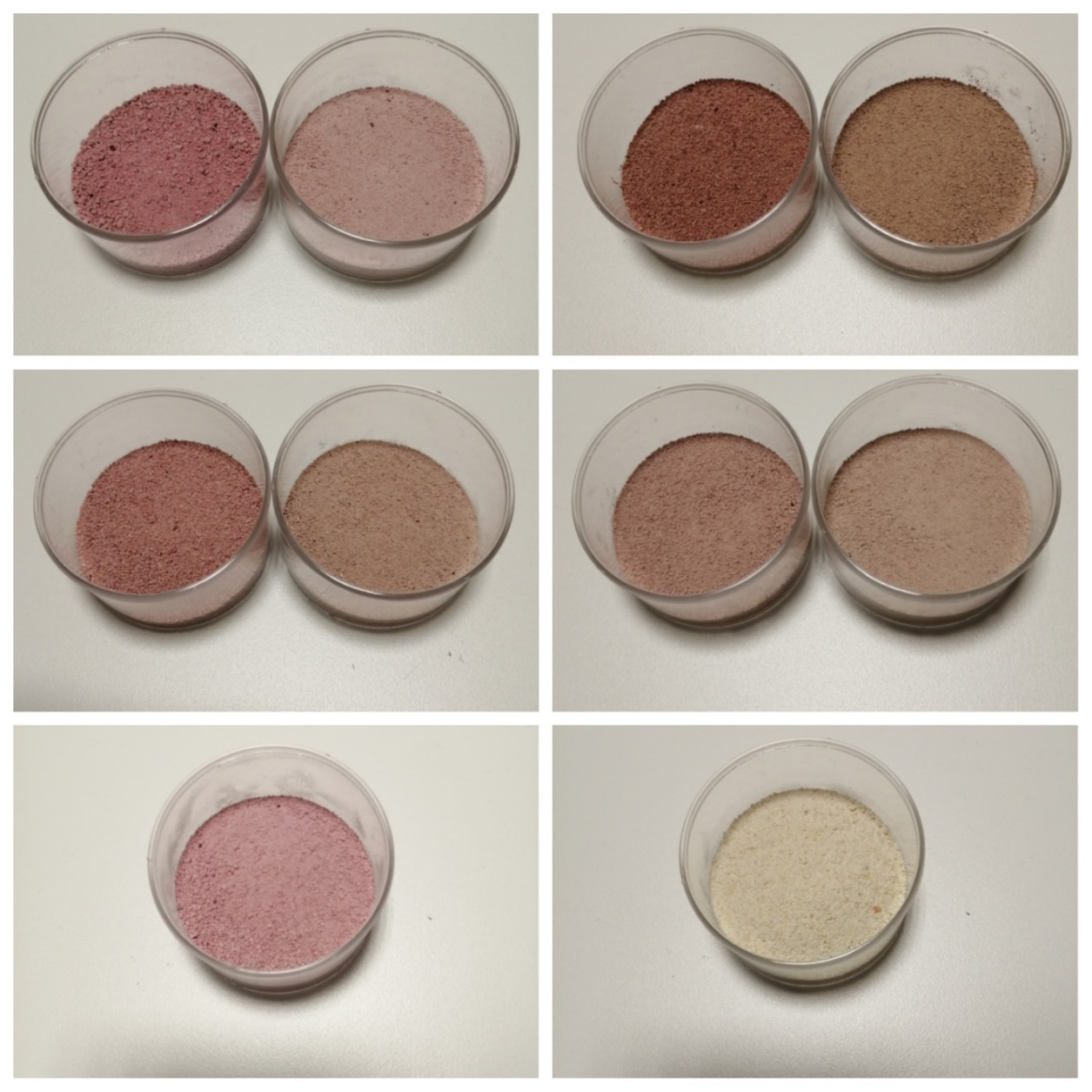
**

**F-BWF**

**N-BWF**

**F-WHBF**

**N-WHBF**

**CCCT**

**CTT**

**F-CF**

**N-CF**

**N-WF**

**F-WF**

**Fig. S1** Cornelian cherry tarhana powders produced with wheat flour (WF, control), wholegrain hull-less barley flour (WHBF), clear flour (CF) and buckwheat flour (BWF). N: non-fermented; F: fermented; CCCT: Commercial cornelian cherry tarhana; CTT: Commercial traditional tarhana.

**Text S1.** ***Detailed Information for Elemental Analyses: ICP-MS/MS Device Settings and Conditions***

ICP-MS/MS sample introduction system constituted with Peltier cooled double pass spray chamber, PTFE inert concentric nebulizer and quartz torch with 2.5 mm internal diameter. An octopole reaction-collision cell (ORC) was placed between two quadrupole mass filters namely: Q1 and Q2. The ORC can be vented or pressurized with He (collision gas) for single MS mode analysis, and O_2_ (reaction gas) in tandem (MS/MS) mode analysis. Calcium, S and P were measured in MS/MS mode while the other elements were detected in single MS mode. Detailed operating conditions of the ICP-MS/MS system are given in **Table S1** as follows: Radiofrequency power: 1550 W, carrier gas flow: 0.50 L/min, make-up gas flow: 0.10 L/min, spray chamber temperature: 0°C, nebulizer: PFA micro-flow (200 μL/min), sampling cone: nickel (1 mm diameter), skimmer cone: nickel (0.8 mm diameter), injector: sapphire, spray chamber: PFA, auto-sampler: Agilent ASX 500, sample uptake time: 50 s, rinse time: 20 s, cell gas: O_2_, gas flow rate: 30% in MS/MS mode and octapole relative frequency: 190 V.

Mixture standards (standard solution of ICP-MS-68A-A with 48 elements, standard solution of ICP-MS-68A-B with 13 elements and standard solution of ICP-MS-68A-C with 7 elements, each was 100 μg/mL) were provided from High-Purity Standards (North Charleston, SC, USA) and used for preparation of multi-element standard stock solutions. Preparation of sulphur standards was performed by using a solid salt, Na_2_SO_4_ (Sigma-Aldrich, MO, USA) dissolved in 2% of HNO_3_. The list of elements with analysis modes and selected isotopes is given in **Table S2**.

For each set of digestion, a laboratory acid blank was prepared. All blanks were prepared and analysed with same method as the food samples for pursuing any contamination during sample handling and preparation steps. Calibration of ICP-MS/MS was performed for six levels (0, 25, 50, 100, 250 and 500 μg/L). Before analysis, ICP-MS/MS was tuned with a tuning solution containing the quantities of 7 (Li), 89 (Y) and 205 (Tl). Tune operation was used for optimization of torch position, sample depth, extraction/ion focus lense voltages, omega lens voltage, quadrupole settings, electron multiplier settings, oxide and doubly charge ratios. Oxides and doubly charge ratios in ICP-MS/MS were always kept below 1% and 2%, respectively.

Standard reference materials (SRM 1567a, wheat flour and SRM 1573a, tomato leaves) obtained from National Institute of Standards and Technology (NIST; Gaithersburg, MD, USA) were used for checking the accuracy of the analytical technique. SRM 1567a was digested and analysed. Recoveries of the Ca, Mg, K, S, Al, Cu, Fe, Mn, Mo, Rb, Na, and Zn were above 95% by the repeated analyses. The linearity of the calibration curves was evaluated by analysing a standard reference material (NIST SRM 1640a, trace elements in natural water). ICP-MS/MS was re-calibrated when the SRM 1640a results were deviated more than 10% of the certified value (**Table S3**). Instrumental detection limit (LODs) and average blank concentrations are given in **Table S4**.

**Table S1.** Device conditions and tuning parameters for ICP-MS/MS system

| **Parameter** | **Value** | **Parameter** | **Reaction cell** | | |  |
| --- | --- | --- | --- | --- | --- | --- |
| RF Power (W) | 1550 | Measurement mode | On-mass | Mass shift | |  |
| Sampling depth (mm) | 8.0 | Cell gas | O_2_ | | |  |
| Carrier gas flow (L min^-1^) | 0.50 | Gas flow rate (%) | 30 | | |  |
| Make up gas flow (L min^-1^) | 0.10 | Octapole Bias (V) | -18 | | |  |
| Spray chamber temp. (°C) | 0 | Energy Dicrimination (V) | -7 | | -7 |  |
| Nebulizer | PFA MicroFlow  (200 μL min^-1^) | Octapole RF (V) | 190 | | |  |
| Sampling cone (orifice dia., mm) | Nickel (1) |  |  | | |  |
| Skimmer cone (orifice dia., mm) | Nickel (0.8) |  |  | | |  |
| Injector | Sapphire |  |  | | |  |
| Spray Chamber | PFA |  |  | | |  |
| Auto sampler | Agilent ASX 500 |  |  | | |  |
| Rinse time (s) | 20 |  |  | | |  |
| Sample uptake time (s) | 50 |  |  | | |  |

**Table S2.** Selected isotopes and analysis modes

| **Isotope/Element** | **Device mode** | **Mass shift** | **Isotope/Element** | **Device mode** | **Mass shift** |
| --- | --- | --- | --- | --- | --- |
| ^7^Li | He |  | ^95^Mo | He |  |
| ^9^Be | No gas |  | ^101^Ru | No gas |  |
| ^11^B | No gas |  | ^103^Rh | No gas |  |
| ^23^Na | He |  | ^105^Pd | No gas |  |
| ^24^Mg | He |  | ^107^Ag | No gas |  |
| ^27^Al | He |  | ^111^Cd | No gas |  |
| ^31^P | O_2_ (MS/MS) | 31→47 (PO^+^) | ^115^In | No gas |  |
| ^32^S | O_2_ (MS/MS) | 32→48 (SO^+^) | ^118^Sn | No gas |  |
| ^39^K | He |  | ^121^Sb | No gas |  |
| ^44^Ca | O_2_ (MS/MS) |  | ^125^Te | No gas |  |
| ^47^Ti | He |  | ^133^Cs | No gas |  |
| ^51^V | He |  | ^137^Ba | No gas |  |
| ^52^Cr | He |  | ^139^La | No gas |  |
| ^55^Mn | He |  | ^140^Ce | No gas |  |
| ^56^Fe | He |  | ^146^Nd | No gas |  |
| ^59^Co | He |  | ^147^Sm | No gas |  |
| ^60^Ni | He |  | ^169^Tm | No gas |  |
| ^63^Cu | He |  | ^175^Lu | No gas |  |
| ^66^Zn | He |  | ^178^Hf | No gas |  |
| ^69^Ga | He |  | ^181^Ta | No gas |  |
| ^72^Ge | He |  | ^182^W | No gas |  |
| ^75^As | He |  | ^193^Ir | No gas |  |
| ^78^Se | He |  | ^195^Pt | No gas |  |
| ^79^Br | He |  | ^197^Au | No gas |  |
| ^85^Rb | He |  | ^205^Tl | No gas |  |
| ^88^Sr | He |  | ^208^Pb | No gas |  |
| ^89^Y | He |  | ^209^Bi | No gas |  |
| ^90^Zr | He |  | ^238^U | No gas |  |
| ^93^Nb | He |  |  |  |  |

**Table S3.** Certified Reference Material (CRM) mineral content (mg/g) for analysis quality reliability assessment, values found in the analysis and recovery rates (%).

| Elements | NIST CRM 1567a (Wheat Flour) | | | NIST CRM 1573a (Tomato Leaves) | | |
| --- | --- | --- | --- | --- | --- | --- |
|  | **Certified (mg/g)** | **Analysed (mg/g)** | **Recovery (%)** | **Certified (mg/g)** | **Analysed (mg/g)** | **Recovery (%)** |
| Na | 6.1±0.8 | 5.9±0.09 | 95.7±1.5 | 136.1±3.7 | 131.7±1.5 | 96.8±1.1 |
| Mg | 400±20 | 406±19 | 101.5±4.9 | NA | NA | NA |
| K | 1330±30 | 1333±19 | 100.2±1.45 | 26760±480 | 25370.3±481.9 | 94.6±1.8 |
| S | 1650±20 | 1610±38 | 97.4±2.3 | NA | NA | NA |
| Ca | 191±4 | 177±9 | 92.7±0.55 | 50450±550 | 46517.2±993 | 92.3±1.9 |
| B | NA | NA | NA | 33.1±0.42 | 31.6±0.39 | 95.4±1.1 |
| Al | 5.7±1.3 | 5.5±0.11 | 96.2±1.9 | 598.4±7.1 | 581.1±7.1 | 97.2±1.2 |
| Mn | 9.4±0.9 | 9.2±0.2 | 97.9±2.3 | 246.3±7.1 | 234.5±4.2 | 95.3±1.7 |
| Rb | 0.68±0.03 | 0.646±0.02 | 95±2.7 | 14.8±0.31 | 13.72±0.24 | 92.5±1.6 |
| Mo | 0.48±0.03 | 0.44±0.02 | 92.1±3.3 | NA | NA | NA |
| Sm | NA | NA | NA | NA | NA | NA |
| Cr | NA | NA | NA | 1.9±0.034 | 1.85±0.03 | 93.2±1.8 |
| Fe | 14.1±0.5 | 14.6±1.07 | 103.8±7.6 | 367.5±4.3 | 352.6±5.1 | 96.0±1.4 |
| Ni | NA | NA | NA | 1.58±0.04 | 1.49±0.01 | 94.2±0.7 |
| Cu | 2.1±0.2 | 2.2±0.04 | 104.8±1.9 | 4.7±0.14 | 4.60±0.01 | 97.9±0.3 |
| Zn | 11.6±0.4 | 11.4±0.5 | 97.9±3.9 | 30.9±0.55 | 28.7±0.24 | 92.8±0.8 |
| Sn | 0.0033 | 0.0032±0,0001 | 96.7±2.7 | NA | NA | NA |
| Pb | 0.02 | 0.019±0,001 | 95±4.2 | NA | NA | NA |
| Ti | NA | NA | NA | NA | NA | NA |
| *Mean±Std Dev, (n=10). NA; Not Available* | | | | | |  |

**Table S4.** Instrumental detection limits and blank concentrations (µg/L)

| Elements | LOD (µg/L) | Blank Concentration (µg/L) |
| --- | --- | --- |
| Na | 1,35299 | 10,51753 |
| Mg | 0,42887 | 1,370056 |
| K | 2,19390 | 29,15813 |
| Ca | 1,63271 | 3,495738 |
| S | 4,98996 | 17,67958 |
| Li | 0,01932 | 0,345838 |
| B | 0,22698 | 1,239638 |
| Al | 0,35647 | 1,601959 |
| Mn | 0,01530 | 0,033486 |
| Sr | 0,03053 | 0,040286 |
| Mo | 0,02929 | 0,197233 |
| In | 0,01796 | 0,074935 |
| Sm | 0,01405 | 0,009711 |
| Cr | 0,01004 | 0,18496 |
| Fe | 0,33128 | 1,678151 |
| Ni | 0,02788 | 0,080512 |
| Cu | 0,15148 | 0,539118 |
| Zn | 0,93298 | 4,073986 |
| Sn | 0,52462 | 1,813343 |
| Pb | 0,03752 | 0,180693 |
